# Metagenomic Screening for Aromatic Compound-Responsive Transcriptional Regulators

**DOI:** 10.1371/journal.pone.0075795

**Published:** 2013-09-30

**Authors:** Taku Uchiyama, Kentaro Miyazaki

**Affiliations:** 1 Bioproduction Research Institute, National Institute of Advanced Industrial Science and Technology, Tsukuba, Ibaraki, Japan; 2 Bioproduction Research Institute, National Institute of Advanced Industrial Science and Technology, Sapporo, Hokkaido, Japan; 3 Department of Medical Genome Sciences, Graduate School of Frontier Sciences, the University of Tokyo, Hokkaido, Japan; Shenzhen Institutes of Advanced Technology, China

## Abstract

We applied a metagenomics approach to screen for transcriptional regulators that sense aromatic compounds. The library was constructed by cloning environmental DNA fragments into a promoter-less vector containing green fluorescence protein. Fluorescence-based screening was then performed in the presence of various aromatic compounds. A total of 12 clones were isolated that fluoresced in response to salicylate, 3-methyl catechol, 4-chlorocatechol and chlorohydroquinone. Sequence analysis revealed at least 1 putative transcriptional regulator, excluding 1 clone (CHLO8F). Deletion analysis identified compound-specific transcriptional regulators; namely, 8 LysR-types, 2 two-component-types and 1 AraC-type. Of these, 9 representative clones were selected and their reaction specificities to 18 aromatic compounds were investigated. Overall, our transcriptional regulators were functionally diverse in terms of both specificity and induction rates. LysR- and AraC- type regulators had relatively narrow specificities with high induction rates (5-50 fold), whereas two-component-types had wide specificities with low induction rates (3 fold). Numerous transcriptional regulators have been deposited in sequence databases, but their functions remain largely unknown. Thus, our results add valuable information regarding the sequence–function relationship of transcriptional regulators.

## Introduction

Bacteria that degrade aromatic compounds are widely distributed in the environment and are important for breaking down both natural and xenobiotic compounds. Attempts have been made to screen for microorganisms that degrade aromatic compounds [1–3], as well as genes responsible for degradation [1,3–7]. These studies revealed that the majority of reported bacterial aromatic degradation processes are aerobic [8] and comprise a series of enzymes that are usually categorized as either ‘upper’- or ‘lower’-pathway enzymes [9]. In the upper pathway, aromatic compounds are transformed into aromatic vicinal diols, which is performed by a monoxygenase or dioxygenase [10]. The aromatic vicinal diols are then converted to dihydroxy compounds by dihydrodiol dehydrogenase in the upper pathway. In the lower pathway, the resulting dihydroxylated aromatic compounds are transformed into ring-cleavage products by either extradiol dioxygenases or intradiol dioxygenases. The subsequent metabolic steps are referred to as meta- or ortho- pathways. The ring-cleavage products are further degraded into compounds that can enter the tricarboxylic acid cycle. These studies revealed large diversity in the degradation pathways and enzymes that depend on compounds and microbial origins. The advent of metagenomic approaches has revealed an even higher degree of diversity [11–15]. We have previously targeted extradiol dioxygenases, which convert colorless catecholic compounds to yellowish ring-opened products, to screen degradation pathways and identify novel enzymes belonging to new subfamilies [11], as well as novel arrangements in the degradation pathway genes [16,17].

In addition to these enzyme-encoding genes involved in aromatic compound degradation, we have previously screened metagenomic libraries for regulatory elements that sense aromatic compounds [18]. Using the fluorescence-based reporter assay system designated SIGEX (Substrate-induced Gene Expression), we identified transcriptional regulators that sense benzoate (8 clones) and naphthalene (2 clones) [18]. In this study, we applied SIGEX to screen for transcriptional regulators in the same library using salicylate, 3-methyl catechol, 4-chlorocatechol and chlorohydroquinone as inducers. These compounds can be used to screen various degradation pathways for aromatic compounds and should provide us with a comprehensive view of the transcriptional regulators responsible for aromatic compound degradation.

## Materials and Methods

### Reagents

Restriction enzymes and DNA ligase were purchased from Takara Bio (Shiga, Japan). Aromatic compounds were purchased from Tokyo Chemical Industry (Tokyo, Japan). Media and agar were purchased from BD Diagnostics (Sparks, MD).

### Bacterial strains, media and growth conditions

*E. coli* JM109 (*recA1 endA1 gyrA96 thi-1 hsdR17 supE44 relA1* Δ(*lac-proAB*) /F’ [*tra36 proAB* ^+^ *lacI* ^q^ *lacZ*∆*M15*]) was used as a host in this study. Cells were grown in LB (10-g peptone, 5-g yeast extract and 5-g NaCl per liter) or dLB (1-g tryptone, 0.5-g yeast extract, 1-g NaCl, 2-g maltose and 10 mL of 1 M MgSO_4_ per liter). When grown on agar plates, LB medium was solidified by adding 1.5% (w/v) agar. Ampicillin (Amp) was added to the medium at 100 µg/ml, when necessary.

### Library construction and screening

The metagenomic library was constructed using environmental DNA extracted from groundwater contaminated with crude oil, as described previously [18]. The library consisted of approximately 152,000 clones with an average insert size of ~7 kb. The library cells were grown in the presence of 0.5 mM isopropyl-β-D-thiogalactopyranoside (IPTG), sorted on a FACSVantage SE (Becton Dickinson, Franklin Lakes, NJ) fluorescence activated cell sorter, and non-fluorescent cells were recovered. These cells were then grown in the presence of 2 mM induction compound, after which fluorescent cells were recovered and singly isolated by growing on LB/Amp agar plates at 37°C overnight. A total of 96 colonies were picked from the plates, resuspended in separate wells of 96-deep-well plates containing 1 mL of LB/Amp, and grown at 37°C overnight. An aliquot of the cells was transferred to 1 mL of fresh dLB/Amp and grown with vigorous shaking at 1,200 rpm at 37°C for 6 h. Cells were divided into 0.5-mL aliquots in 96-deep-well plates containing 0.5 mL of fresh dLB/Amp with or without 2 mM of test compound. Cells were then grown at 37°C for 24 h by shaking the 96-deep-well plates at 1,200 rpm, pelleted by centrifugation (1,500 *g*, 5 min), washed with distilled water, and resuspended in 200 µL of distilled water. The cell suspension (100 µL) from each well was transferred to the wells of a black, clear-bottomed 96-well plate. GFP fluorescence was measured on a SPECTRAmax Gemini XS (Molecular Devices, Sunnyvale, CA) spectrofluorimeter at excitation and emission wavelengths of 488 and 520 nm, respectively. Cell density (OD_600_) was measured on a VERSAMax (Molecular Devices) UV-Vis microplate reader. Fluorescence was normalized to the cell density (OD_600_ of 1.0). Positive clones showing an induction rate (ratio of fluorescence intensity +inducer/-inducer) higher than 3.0 were collected. These clones were then subjected to restriction fragment length polymorphism (RFLP) analysis using *Eco*RI or *Hin*dIII to check for possible duplication.

### DNA sequencing and sequence analysis

DNA sequencing was performed using the BigDye Terminator v3.1 Cycle Sequencing Kit (Applied Biosystems, Foster City, CA) and an ABI3730XL sequencer (Applied Biosystems). Open reading frame analysis was performed using a web-based ORF Finder software at http://www.ncbi.nlm.nih.gov/. Homology searches were performed using BLASTX at http://blast.ncbi.nlm.nih.gov/ with the default parameter settings. Amino acid sequence alignment was performed using the web-based ClustalW version 1.83 software tool at http://clustalw.ddbj.nig.ac.jp/top-j.html, and the results were refined by visual inspection. Neighbor-joining trees were constructed using FigTree, version 1.3.1 (http://tree.bio.ed.ac.uk/software/Figtree/). Other sequence analyses were performed using the GENETYX-MAC version 15.0.0 software package (GENETYX Co., Tokyo, Japan).

### Deletion analysis

**Figure 1:**
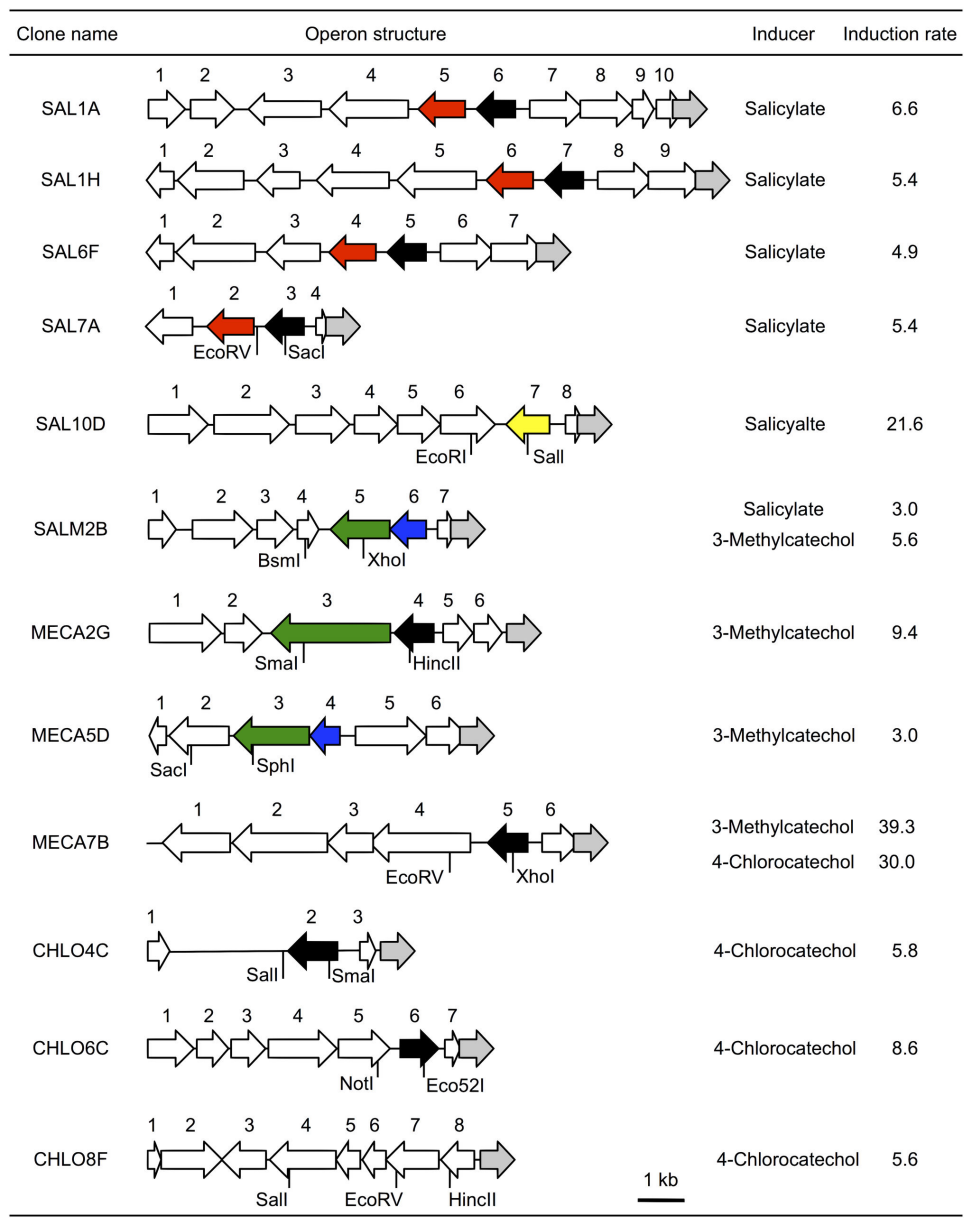
Operonic structures of aromatic compound-responsive clones. Arrows indicate GFP (grey), and putative transcriptional regulators are in black (LysR-type), red (Fis-type), blue (two-component system response regulator), green (two-component system, histidine kinase) and yellow (AraC-type). Restriction sites are those used for construction of deletion derivatives.

Deletion analysis was performed using restriction digestion and self-ligation. Restriction enzymes used for the deletion are shown in Figure 1. The resulting ligation mixture was used to transform *E. coli* JM109 by electroporation.

### Nucleotide sequences accession number

Nucleotide sequences reported in this paper have been submitted to the GenBank/EMBL/DDBJ under accession Nos. AB828163 to AB828174.

## Results and Discussion

### Screening for transcriptional regulators from the metagenomic library

A metagenomic library was constructed using groundwater contaminated with crude oil. In our previous study, we screened the library for transcriptional activators that specifically sensed benzoate and naphthalene [18]. In this study, we screened the same library using different aromatic inducing compounds; namely, salicylate, 3-methylcatechol and 4-chlorocatechol. Salicylate is a key metabolic intermediate in polycyclic aromatic hydrocarbon catabolic pathways [19,20]. 3-Methylcatechol is also a key metabolic intermediate in the o-xylene, *m*-xylene and toluene catabolic pathways [2,21], while 4-chlorocatechol is a key metabolic intermediate of several chlorophenoxyacetic acid herbicides [22]. These compounds play a role in the degradation pathways of various aromatic compounds.

SIGEX was applied to screen the library and yielded 120 positive clones: 54 clones for salicylate, 30 for 3-methylcatechol and 36 for 4-chlorocatechol. The sorting ratio (ratio of the number of fluorescence cells to total cells subjected to cell sorting) was 1.4×10^-4^, which was similar to the value (2.3×10^-4^) obtained in our previous experiment using benzoate and naphthalene as inducing compounds [18]. 

Since the library was amplified in liquid medium, RFLP analysis was conducted to remove redundant clones. Based on the restriction pattern, salicylate-inducible clones were divided into five types (SAL1A, SAL1H, SAL6F, SAL7A, SAL10D), 3-methylcatechol-inducible clones into two types (MECA2G, MECA5D), and 4-chlorocatechol-inducible clones into three types (CHLO4C, CHLO6C, CHLO8F). These clones were tested for cross reactivity, which revealed that SALM2B had dual specificity for salicylate and 3-methylcatechol, and MECA7B to 3-methylcatechol and 4-chlorocatechol. In total, we recovered 12 types of clone (Figure 1, Table S1). The maximum induction rate was observed for MECA7B when 3-methylcatechol was used as an inducer (39.3-fold induction). Minimum induction rates were observed for SALM2B (3.0-fold induction with salicylate) and MECA5D (3.0-fold induction with 3-methylcatechol). Positive clones did not degrade the inducible compounds based on HPLC analysis.

### Sequence analysis of the metagenomic fragments

To determine whether selected clones contained transcriptional regulators, we performed DNA sequencing analysis of the 12 positive clones (Figure 1, Table S1). We found that all but CHLO8F carried ORFs classified into known families of transcriptional regulators (Figure 1, Table S2).

Eight ORFs belonged to LysR-type (SAL1A_ORF6, SAL1H_ORF7, SAL6F_ORF5, SAL7A_ORF3, MECA2G_ORF4, MECA7B_ORF5, CHLO4C_ORF2, CHLO6C_ORF6) and four belonged to Fis-type (SAL1A_ORF5, SAL1H_ORF6, SAL6F_ORF4 and SAL7A_ORF2). SALM2B and MECA5D contained ORFs homologous to two-component signal transduction systems, response regulators (SALM2B_ORF6, MECA5D_ORF4) and histidine kinase (SALM2B_ORF5, MECA5D_ORF3). MECA2G also contained an ORF homologous to receptor histidine kinase (MECA2G_ORF3), but lacked the response regulator gene. SAL10D contained an AraC-type transcriptional regulator (SAL10D_ORF7). With one exception, no ORFs were found in CHLO8F related to transcriptional regulators.

Regarding enzyme-encoding genes, SAL1A, SAL1H, SAL6F and SAL7A contained ORFs that were homologous to salicylate oxygenase components (SAL1A_ORF8 to 10, SAL1H_ORF9, SAL6G_ORF7) and 2,4-dinitrotoluene dioxygenase components (SAL1A_ORF7, SAL1H_ORF8, SAL6F_ORF6, and SAL7A_ORF4). SAL10D contained a monooxygenase-like sequence (SAL10D_ORF8) and MECA5D contained a multicopper oxidase-like sequence (MECA5D_ORF6), both of which may be involved in aromatic-hydrocarbon transformation.

Notably, 4 clones (SAL1A, SAL1H, SAL6F and SAL7A) shared high DNA sequence similarities. SAL1A_ORF3 and SAL1A_ORF8 were 99% identical to SAL1H_ORF4 and SAL1H_ORF9, respectively. Despite this high similarity, flanking ORFs SAL1A_ORF2 and SAL1H_ORF3 lacked homology. ORF3 to ORF6 in SAL6F were identical to ORF1 to ORF4 of SAL7A, suggesting that the 2 metagenomic fragments were derived from the same origin. DNA sequences of SAL1H and SAL6F were closely related, but SAL6F contained an additional gene (ORF3) between ORF5 (glucose-methanol-choline oxidoreductase) and ORF6 (Fis family transcriptional regulator) in SAL1H. SAL1H_ORF6 and SAL1H_ORF9 were 86% identical to SAL6F_ORF4 and SAL6F_ORF7, respectively. SAL1H_ORF5 and SAL6F_ORF2 shared 90% identities, but SAL1H_ORF4 and SAL6F_ORF1 were non-homologous. Because SAL7A shared high (86 to 100%) identities with SAL1A, SAL1H, and SAL6F, we used SAL7A as a representative clone among salicylate-inducible clones for functional analysis.

### Functional identification of regulatory elements by deletion analysis

**Figure 2:**
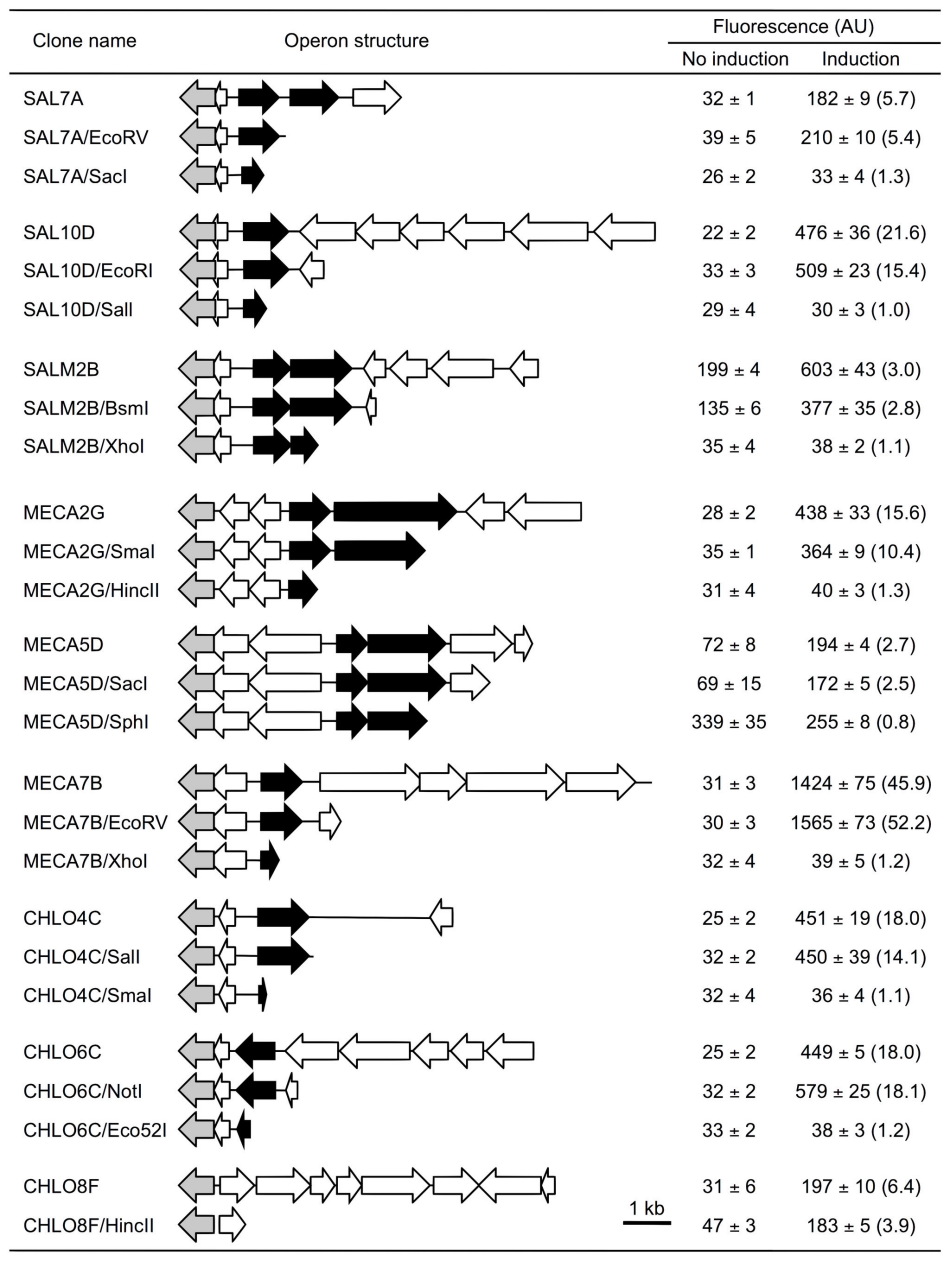
Deletion analysis of aromatic compounds-responsive clones. Inducing compounds are salicylate for SAL7A, SAL10D, SALM2B and their deletion derivatives, 3-methylcatechol for MECA2G, MECA5D, MECA7B and their deletion derivatives, and 4-chlorocatechol for CHLO4C, CHLO6C, CHLO8F and their deletion derivatives, respectively. Fluorescence intensity was normalized to cell density. All values are the means of five measurements. Bracket values indicate induction rates (fold increased). Arrows indicate GFP (grey), and putative transcriptional regulators are in black (LysR-type), red (Fis-type), blue (two-component system response regulator), green (two-component system, histidine kinase) and yellow (AraC-type).

To functionally identify the ORFs responsible for transcriptional activation, we created a series of deletion derivatives that were subjected to induction tests (Figure 2).

Partial deletion of LysR-type regulators, SAL7A_ORF3 (pSAL7A/SacI), MECA2G_ORF4 (pMECA2G/HincII), MECA7B_ORF5 (pMEA7B/XhoI), CHLO4C_ORF2 (pCHLO4C/SmaI) and CHLO6C_ORF6 (pCHLO6C/Eco52I) abolished their functions based on the disappearance of GFP fluorescence in the presence of test compounds. Partial deletion of AraC-type SAL10D_ORF7 (pSAL10D/SalI) and receptor histidine kinase homolog SALM2B_ORF5 (pSALM2B/XhoI) also resulted in the loss of the inducing activity. Therefore, transcriptional regulators included in these clones (SAL7A, SAL10D, SALM2B, MECA2G, MECA7B, CHLO4C and CHLO6C) are responsible for compound-specific transcriptional activation.

In contrast, the deletion derivative of a receptor histidine kinase homolog MECA5D_ORF3 (pMECA5D/SphI) constitutively expressed GFP. In this variant, responsive regulator MECA5D_ORF4, which is located adjacent to the histidine kinase, might have lost its activity, i.e., transcriptional repression, due to the inactivation of the cognate kinase.

Regarding CHLO8F, a series of deletion mutants (pCHLO8F/SalI, pCHLO8F/EcoRV and pCHLO8F/HincII) was constructed, but all of them had induction properties similar to the original clone. Most of the ORFs included in pCHLO8F were flagellar proteins (Table S1), and we could not identify protein elements responsible for the specific induction by 4-chlorocatechol.

### Induction specificity of the metagenomic transcriptional regulators

**Figure 3:**
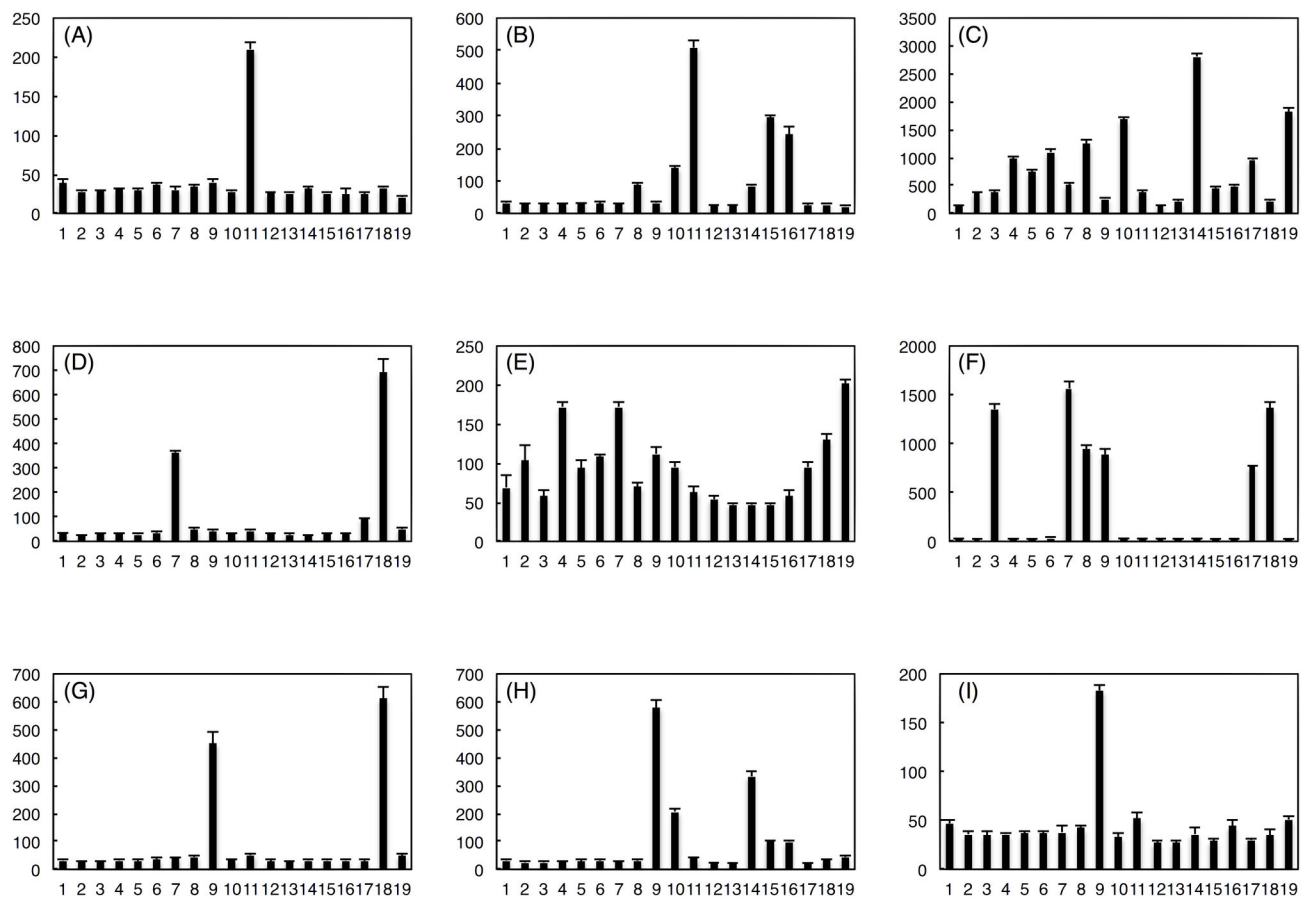
Induction specificity of metagenomically retrieved transcriptional regulators. Clones: **A**: pSAL7A/EcoRV, **B**: pSAL10D/EcoRI, **C**: pSALM2B/BsmI, **D**: pMECA2G/SmaI, **E**: pMECA5D/SacI, **F**: pMECA7B/EcoRV, **G**: pCHLO4C/SalI, **H**: pCHLO6C/NotI, and **I**: pCHLO8F/HincII. Induction compound: 1, none; 2, phenol; 3, catechol; 4, o-cresol; 5, *m*-cresol; 6, *p*-cresol; 7, 3-methylcatechol; 8, 4-methylcatechol; 9, 4-chlorocatechol; 10, benzoate; 11, salicylate; 12, 3-hydroxybenzoate; 13, 4-hydroxybenzoate; 14, 2-chlorobenzoate; 15, 3-chlorobenzoate; 16, 4-chlorobenzoate; 17, hydroquinone; 18, chlorohydroquinone; and 19, 4-chlororesorcinol. Fluorescence intensity was normalized to cell densities. Values represent the means of five measurements.

We next used the nine deletion derivative clones to test the induction specificity towards 18 aromatic compounds. Induction was performed in dLB medium in the presence and absence of 2 mM test compound. As shown in Figure 3, our transcriptional regulators retrieved from the metagenome are functionally diverse both in specificity and induction rate.

Among the nine tested clones, four (SAL7A/EcoRV, CHLO8F/HincII, CHLO4C/SalI and MECA2G/SmaI) showed narrow specificity. SAL7A/EcoRV (panel A, LysR-type) was extremely specific to salicylate (5.4 fold relative to no induction), while CHLO8F/HincII (panel I, transcriptional regulator unidentified) was specific to 4-chlorocatechol (3.9 fold) among the 18 test compounds (phenol, catechol, o-cresol, *m*-cresol, *p*-cresol, 3-methylcatechol, 4-methylcatechol, 4-chlorocatechol, benzoate, salicylate, 3-hydroxybenzoate, 4-hydroxybenzoate, 2-chlorobenzoate, 3-chlorobenzoate, 4-chlorobenzoate, hydroquinone, chlorohydroquinone and 4-chlororesorcinol). CHLO4C/SalI (panel G, LysR-type) was specific to 4-chlorocatechol (14.1 fold) and chlorohydroquinone (19.1 fold), both with high induction rates. MECA2G/SmaI (panel D, LysR-type) recognized 3-methylcathechol (10.4 fold) and chlorohydroquinone (19.8 fold). Overall, these clones showed relatively high induction rates.

Five clones (SAL10D/EcoRI, SALM2B/BsmI, MECA5D/SacI, MECA7B/EcoRV, CHLO6C/NotI, and SALM2B/BsmI) showed broad specificities to various compounds, the induction rates of which varied. SAL10D/EcoRI (panel B, AraC-type) recognized 4-methylcatechol (2.7 fold), benzoate (4.2 fold), salicylate (15.4 fold), 2-chlorobenzoate (2.6 fold), 3-chlorobenzoate (8.9 fold) and 4-chlorobenzoate (7.4 fold). SALM2B/BsmI (panel C, two-component-type) showed the most relaxed, intemperate specificity, which recognized phenol (2.8 fold), catechol (2.8 fold), *o-*cresol (7.3 fold), *m*-cresol (5.5 fold), *p*-cresol (8.1 fold), 4-methycatechol (3.8 fold), 4-methylcatechol (9.2 fold), benzoate (12.4 fold), salicylate (2.8 fold), 2-chlorobenzoate (20.1 fold), 3-chlorobenzoate (3.3 fold), 4-chlorobenzoate (3.6 fold), hydroquinone (7.1 fold) and 4-chlororesorcinol (13.4 fold). MECA5D/SacI (panel E, two-component-type) recognized relaxed *o-*cresol (2.5 fold), 3-methylcatechol (2.5 fold) and 4-chlororesorcinol (2.9 fold). MECA7B/EcoRV (panel F) recognized catechol (45.1 fold), 3-methylcatechol (52.2 fold), 4-methylcatechol (31.5 fold), 4-chlorocatechol (29.5 fold), hydroquinone (25.6 fold) and chlorohydroquinone (45.3 fold) as induction compounds. CHLO6C/NotI (panel H, LysR-type) recognized 4-chlorocatechol (18.1 fold), benzoate (6.5 fold), 2-chlorobenzoate (10.4 fold), 3-chlorobenzoate (3.2 fold) and 4-chlorobenzoate (3.1 fold). 

In general, LysR-type regulators had relatively narrow specificities with high induction rates, while two-component-types had wide specificities with low induction rates.

### Phylogenetic analysis of the metagenomically retrieved transcriptional regulators

The phylogenetic relationship of metagenomic and functionally characterized LysR-type transcriptional regulators is shown in Figure S1. Our metagenomic LysR-type transcriptional regulators (SAL7A_ORF3, MECA2G_ORF4, MECA7B_ORF5, CHLO4C_ORF2 and CHLO6C_ORF6) were sparsely distributed in the phylogenetic tree. SAL7A_ORF3 shared 61 to 78% primary amino acid sequence identity with a LysR-type transcriptional regulators related to naphthalene and salicylate degradation operon regulator NahR (A31382) from *Pseudomonas* *putida* strain G7 plasmid NAH7 [23], salicylate degradation operon regulator NahR (AAD02145) from *P* *. stutzeri* strain AN10 [24], naphthalene degradation operon regulator NagR (Q9EXL7) from *Ralstonia* sp. strain U2 [25], nitrobenzene degradation pathway operon regulator NbzR (Q7WT52) from *Comamonas* sp. strain JS765 and 2-nitrotoluene degradation pathway operon regulator NtdR (AAP70492) from *Acidovorax* sp. strain JS42 [26]. The other four transcriptional regulators (CHLO4C_ORF2, CHLO6C_ORF6, MECA2G_ORF4 and MECA7B_ORF5) lacked similarity to known LysR-type regulators involved in aromatic hydrocarbon degradation. In fact, the latter four transcriptional regulator genes were not flanked by genes involved in aromatic degradation. Thus, they may not be related to aromatic compound degradation in nature.

The phylogenetic relationship of metagenomics and functionally characterized AraC-type transcriptional regulators is shown in Figure S2. SAL10D_ORF7 had no significant similarity to known AraC-type transcriptional regulators of aromatic hydrocarbon degradation pathway operons.

The phylogenetic relationship of metagenomic and functionally characterized two-component regulatory system-type transcriptional regulators is shown in Figure S3. Some toluene degradation operons and styrene degradation operons are regulated by two-component signal transduction systems. Their response regulators belong to the NarL-like helix-turn-helix (HTH) family [27], but SALM2B_ORF6 and MECA5D_ORF4 belonged to OmpR-like HTH family, which lack homology to known regulators of aromatic compound degradation. Among the nearest neighbors, SALM2B_ORF6 shared 53% amino acid sequence identity with PmrA (Q02FP6) and SALM2B_ORF5 shared 30% identity with a PmrB (Q02FP5). The PmrA-PmrB system is activated by cationic antimicrobial peptides, which are involved in the regulation of resistance to polymyxin B and cationic antimicrobial peptides in *P. aeruginosa* [28]. However, it is not known whether this system is regulated by aromatic compounds.

MECA5D_ORF4 (response regulator) shared 72% amino acid sequence identity with CopR (1909226A) and MECA5D_ORF3 (histidine kinase) shared 37% amino acid sequence identity with CopS (1909226B). The CopR-CopS system is involved in the regulation of copper resistance in *P. syringae* [29]. CopR directly regulates the copper resistance operon and CopS is activated by high copper concentrations, but how this system responds to aromatic compounds remains unknown.

## Conclusion

Using reporter assay-based screening of a metagenomic library, we successfully identified transcriptional regulators with different compound specificities and induction rates. The majority of these compounds were classified into known regulator types, including LysR, AraC, and two-component. In general, LysR-type regulators had relatively narrow specificities with high induction rates, while two-component-types had wide specificities with low induction rates. A large number of transcriptional regulators have been deposited in sequence databases, but their functions (i.e., compound specificity and induction rate) remain unknown. Our results increase our understanding of the sequence–function relationship of transcriptional regulators.

## Supporting Information

Figure S1 **Phylogenetic relationship between functionally characterized LysR-type transcriptional regulators and our metagenomically retrieved homologues.** Shaded clones are known to be involved in degradation of aromatic compounds.
(PPTX)

Figure S2 **Phylogenetic relationship between functionally characterized AraC-type transcriptional regulators and our metagenomically retrieved homologues.** Shaded clones are known to be involved in degradation of aromatic compounds.
(PPTX)

Figure S3 **Phylogenetic relationship between functionally characterized two component -type transcriptional regulators and our metagenomically retrieved homologues.** **A**: Response regulator domain; **B**: histidine kinase domain. Shaded clones are known to be involved in the degradation of aromatic compounds.
(PPTX)

Table S1 **Plasmids used in this study.**
(DOCX)

Table S2 **List of ORFs in metagenomically retrieved clones that fluoresced in response to aromatic compounds.** Putative transcriptional regulators are shown in boldface and those responsible for compound-specific regulation are underlined.
(DOCX)

## References

[1] Esteve-Núñez A, Caballero A, Ramos JL (2001) Biological degradation of 2,4,6-trinitrotoluene. Microbiol Mol Biol Rev 65: 335–352. doi:10.1128/MMBR.65.3.335-352.2001. PubMed: 11527999. 11527999 10.1128/MMBR.65.3.335-352.2001 PMC99030

[2] Williams PA, Worsey MJ (1976) Ubiquity of plasmids in coding for toluene and xylene metabolism in soil bacteria: evidence for the existence of new TOL plasmids. J Bacteriol 125: 818–828. PubMed: 1254555. 1254555 10.1128/jb.125.3.818-828.1976 PMC236154

[3] Wittich R-M (1998) Degradation of dioxin-like compounds by microorganisms. Appl Microbiol Biotechnol 49: 489–499. doi:10.1007/s002530051203. PubMed: 9650248. 9650248 10.1007/s002530051203

[4] Arai H, Ohishi T, Chang MY, Kudo T (2000) Arrangement and regulation of the genes for meta-pathway enzymes required for degradation of phenol in *Comamonas* *testosteroni* TA441. Microbiology 146: 1707–1715. PubMed: 10878134. 10878134 10.1099/00221287-146-7-1707

[5] Duffner FM, Kirchner U, Bauer MP, Müller R (2000) Phenol/cresol degradation by the thermophilic *Bacillus* *thermoglucosidasius* A7: Cloning and sequence analysis of five genes involved in the pathway. Gene 256: 215–221. doi:10.1016/S0378-1119(00)00352-8. PubMed: 11054550. 11054550 10.1016/s0378-1119(00)00352-8

[6] Furukawa K, Suenaga H, Goto M (2004) Biphenyl dioxygenase: functional versatilities and directed evolution. J Bacteriol 186: 5189–5196. doi:10.1128/JB.186.16.5189-5196.2004. PubMed: 15292119. 15292119 10.1128/JB.186.16.5189-5196.2004 PMC490896

[7] Galvão TC, Mohn WW, de Lorenzo V (2005) Exploring the microbial biodegradation and biotransformation gene pool. Trends Biotechnol 23: 497–506. doi:10.1016/j.tibtech.2005.08.002. PubMed: 16125262. 16125262 10.1016/j.tibtech.2005.08.002

[8] Gibson J, Harwood CS (2002) Metabolic diversity in aromatic compound utilization by anaerobic microbes. Annu Rev Microbiol 56: 345–369. doi:10.1146/annurev.micro.56.012302.160749. PubMed: 12142480. 12142480 10.1146/annurev.micro.56.012302.160749

[9] Williams PA, Sayers JR (1994) The evolution of pathways for aromatic hydrocarbon oxidation in *Pseudomonas* . Biodegradation 5: 195–217. doi:10.1007/BF00696460. PubMed: 7765833. 7765833 10.1007/BF00696460

[10] Gibson DT, Parales RE (2000) Aromatic hydrocarbon dioxygenases in environmental biotechnology. Curr Opin Biotechnol 11: 236–243. doi:10.1016/S0958-1669(00)00090-2. PubMed: 10851146. 10851146 10.1016/s0958-1669(00)00090-2

[11] Suenaga H, Ohnuki T, Miyazaki K (2007) Functional screening of a metagenomic library for genes involved in microbial degradation of aromatic compounds. Environ Microbiol 9: 2289–2297. doi:10.1111/j.1462-2920.2007.01342.x. PubMed: 17686025. 17686025 10.1111/j.1462-2920.2007.01342.x

[12] van Hellemond EW, Janssen DB, Fraaije MW (2007) Discovery of a novel styrene monooxygenase originating from the metagenome. Appl Environ Microbiol 73: 5832–5839. doi:10.1128/AEM.02708-06. PubMed: 17644649. 17644649 10.1128/AEM.02708-06 PMC2074922

[13] Brennerova MV, Josefiova J, Brenner V, Pieper DH, Junca H (2009) Metagenomics reveals diversity and abundance of meta-cleavage pathways in microbial communities from soil highly contaminated with jet fuel under air-sparging bioremediation. Environ Microbiol 11: 2216–2227. doi:10.1111/j.1462-2920.2009.01943.x. PubMed: 19575758. 19575758 10.1111/j.1462-2920.2009.01943.x PMC2784041

[14] Sul WJ, Park J, Quensen JF 3rd, Rodrigues JL, Seliger L, et al. (2009) DNA-stable isotope probing integrated with metagenomics for retrieval of biphenyl dioxygenase genes from polychlorinated biphenyl-contaminated river sediment. Appl Environ Microbiol 75: 5501–5506. doi:10.1128/AEM.00121-09. PubMed: 19648381. 19648381 10.1128/AEM.00121-09 PMC2737913

[15] Silva CC, Hayden H, Sawbridge T, Mele P, De Paula SO, et al. (2013) Identification of genes and pathways related to phenol degradation in metagenomic libraries from petroleum refinery wastewater. PLOS ONE 8: e61811. doi:10.1371/journal.pone.0061811. PubMed: 23637911. 23637911 10.1371/journal.pone.0061811 PMC3630121

[16] Suenaga H, Koyama Y, Miyakoshi M, Miyazaki R, Yano H, et al. (2009) Novel organization of aromatic degradation pathway genes in a microbial community as revealed by metagenomic analysis. ISME J 3: 1335–1348. doi:10.1038/ismej.2009.76. PubMed: 19587775. 19587775 10.1038/ismej.2009.76

[17] Suenaga H, Mizuta S, Miyazaki K (2009) The molecular basis for adaptive evolution in novel extradiol dioxygenases retrieved from the metagenome. FEMS Microbiol Ecol 69: 472–480. doi:10.1111/j.1574-6941.2009.00719.x. PubMed: 19566698. 19566698 10.1111/j.1574-6941.2009.00719.x

[18] Uchiyama T, Abe T, Ikemura T, Watanabe K (2005) Substrate-induced gene-expression screening of environmental metagenome libraries for isolation of catabolic genes. Nat Biotechnol 23: 88–93. doi:10.1038/nbt1048. PubMed: 15608629. 15608629 10.1038/nbt1048

[19] Chen SH, Aitken MD (1999) Salicylate stimulates the degradation of high-molecular weight polycyclic aromatic hydrocarbons by *Pseudomonas* *saccharophila* P15. Environ Sci Technol 33: 435–439. doi:10.1021/es993231i.

[20] Meyer S, Moser R, Neef A, Stahl U, Kämpfer P (1999) Differential detection of key enzymes of polyaromatic-hydrocarbon-degrading bacteria using PCR and gene probes. Microbiology 145: 1731–1741. doi:10.1099/13500872-145-7-1731. PubMed: 10439412. 10439412 10.1099/13500872-145-7-1731

[21] Zylstra GJ, McCombie WR, Gibson DT, Finette BA (1988) Toluene degradation by *Pseudomonas* *putida* F1: genetic organization of the *tod* operon. Appl Environ Microbiol 54: 1498–1503. PubMed: 2843094. 2843094 10.1128/aem.54.6.1498-1503.1988 PMC202686

[22] Evans WC, Smith BS, Moss P, Fernley HN (1971) Bacterial metabolisum of 4-chlorophenozyacetate. Biochem J 122: 509–517. PubMed: 5123884. 5123884 10.1042/bj1220509 PMC1176808

[23] You IS, Ghosal D, Gunsalus IC (1988) Nucleotide sequence of plasmid NAH7 gene *nahR* and DNA binding of the *nahR* product. J Bacteriol 170: 5409–5415. PubMed: 2848005. 2848005 10.1128/jb.170.12.5409-5415.1988 PMC211631

[24] Bosch R, Moore ERB, García-Valdés E, Pieper DH (1999) NahW, a novel, inducible salicylate hydroxylase involved in mineralization of naphthalene by *Pseudomonas* *stutzeri* AN10. J Bacteriol 181: 2315–2322. PubMed: 10197990. 10197990 10.1128/jb.181.8.2315-2322.1999 PMC93652

[25] Zhou NY, Fuenmayor SL, Williams PA (2001) *nag* genes of *Ralstonia* (formerly *Pseudomonas*) sp. strain U2 encoding enzymes for gentisate catabolism. J Bacteriol 183: 700–708. doi:10.1128/JB.183.2.700-708.2001. PubMed: 11133965. 11133965 10.1128/JB.183.2.700-708.2001 PMC94927

[26] Lessner DJ, Parales RE, Narayan S, Gibson DT (2003) Expression of the nitroarene dioxygenase genes in *Comamonas* sp. strain JS765 and *Acidovorax* sp. strain JS42 is induced by multiple aromatic compounds. J Bacteriol 185: 3895–3904. doi:10.1128/JB.185.13.3895-3904.2003. PubMed: 12813084. 12813084 10.1128/JB.185.13.3895-3904.2003 PMC161575

[27] Galperin MY (2006) Structural classification of bacterial response regulators: diversity of output domains and domain combinations. J Bacteriol 188: 4169–4182. doi:10.1128/JB.01887-05. PubMed: 16740923. 16740923 10.1128/JB.01887-05 PMC1482966

[28] McPhee JB, Lewenza S, Hancock RE (2003) Cationic antimicrobial peptides activate a two-component regulatory system, PmrA-PmrB, that regulates resistance to polymyxin B and cationic antimicrobial peptides in *Pseudomonas* *aeruginosa* . Mol Microbiol 50: 205–217. doi:10.1046/j.1365-2958.2003.03673.x. PubMed: 14507375. 14507375 10.1046/j.1365-2958.2003.03673.x

[29] Mills SD, Jasalavich CA, Cooksey DA (1993) A two-component regulatory system required for copper-inducible expression of the copper resistance operon of *Pseudomonas* *syringae* . J Bacteriol 175: 1656–1664. PubMed: 8449873. 8449873 10.1128/jb.175.6.1656-1664.1993 PMC203959

