# Supplementary material for: Metagenomic Screening for Aromatic Compound-Responsive Transcriptional Regulators
Source: PLoS One. 2013 Sep 30;8(9):e75795. doi: 10.1371/journal.pone.0075795 (PMC3786939; doi:10.1371/journal.pone.0075795)
Supplement: Figure S1 — Phylogenetic relationship between functionally characterized LysR-type transcriptional regulators and our metagenomically retrieved homologues. Shaded clones are known to be involved in degradation of aromatic compounds. (PPTX) [file pone.0075795.s001.pptx]

## Slide 1
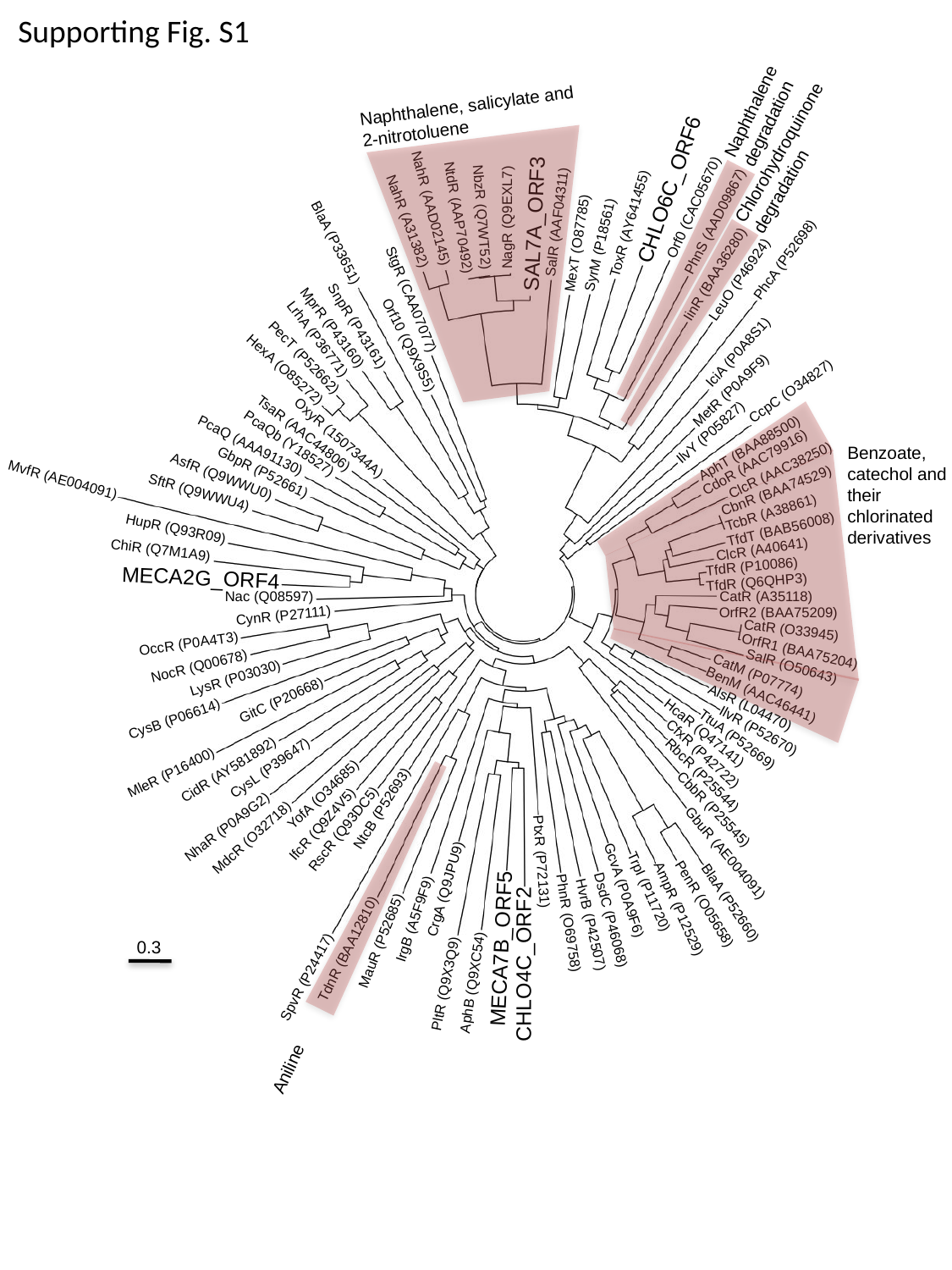

Supporting Fig. S1
Naphthalene degradation
CHLO6C_ORF6
NagR (Q9EXL7)
Orf0 (CAC05670)
SalR (AAF04311)
PhnS (AAD09867)
ToxR (AY641455)
SAL7A_ORF3
MexT (O87785)
NahR (AAD02145)
SyrM (P18561)
NtdR (AAP70492)
NbzR (Q7WT52)
NahR (A31382)
BlaA (P33651)
PhcA (P52698)
IinR (BAA36280)
LeuO (P46924)
StgR (CAA07077)
SnpR (P43161)
MprR (P43160)
IciA (P0A8S1)
LrhA (P36771)
Orf10 (Q9X9S5)
PecT (P52662)
HexA (O85272)
CcpC (O34827)
MetR (P0A9F9)
IlvY (P05827)
TsaR (AAC44806)
AphT (BAA88500)
OxyR (1507344A)
PcaQ (AAA91130)
PcaQb (Y18527)
CdoR (AAC79916)
ClcR (AAC38250)
GbpR (P52661)
CbnR (BAA74529)
AsfR (Q9WWU0)
MvfR (AE004091)
SftR (Q9WWU4)
TcbR (A38861)
TfdT (BAB56008)
HupR (Q93R09)
ClcR (A40641)
ChiR (Q7M1A9)
TfdR (P10086)
MECA2G_ORF4
TfdR (Q6QHP3)
Nac (Q08597)
CatR (A35118)
OrfR2 (BAA75209)
CynR (P27111)
CatR (O33945)
OccR (P0A4T3)
OrfR1 (BAA75204)
NocR (Q00678)
SalR (O50643)
LysR (P03030)
CatM (P07774)
BenM (AAC46441)
GitC (P20668)
AlsR (L04470)
CysB (P06614)
IlvR (P52670)
HcaR (Q47141)
TtuA (P52669)
CidR (AY581892)
CysL (P39647)
CfxR (P42722)
MleR (P16400)
YofA (O34685)
RbcR (P25544)
NtcB (P52693)
RscR (Q93DC5)
IfcR (Q9Z4V5)
NhaR (P0A9G2)
CbbR (P25545)
MdcR (O32718)
GbuR (AE004091)
PtxR (P72131)
CrgA (Q9JPU9)
TrpI (P11720)
BlaA (P52660)
PenR (O05658)
GcvA (P0A9F6)
AmpR (P12529)
IrgB (A5F9F9)
MECA7B_ORF5
DsdC (P46068)
MauR (P52685)
PhnR (O69758)
CHLO4C_ORF2
TdnR (BAA12810)
HvrB (P42507)
SpvR (P24417)
AphB (Q9XC54)
PltR (Q9X3Q9)
Naphthalene, salicylate and 2-nitrotoluene
Chlorohydroquinone degradation
Benzoate, catechol and their chlorinated derivatives
0.3
Aniline
