# Supplementary material for: Metagenomic Screening for Aromatic Compound-Responsive Transcriptional Regulators
Source: PLoS One. 2013 Sep 30;8(9):e75795. doi: 10.1371/journal.pone.0075795 (PMC3786939; doi:10.1371/journal.pone.0075795)
Supplement: Figure S2 — Phylogenetic relationship between functionally characterized AraC-type transcriptional regulators and our metagenomically retrieved homologues. Shaded clones are known to be involved in degradation of aromatic compounds. (PPTX) [file pone.0075795.s002.pptx]

## Slide 1
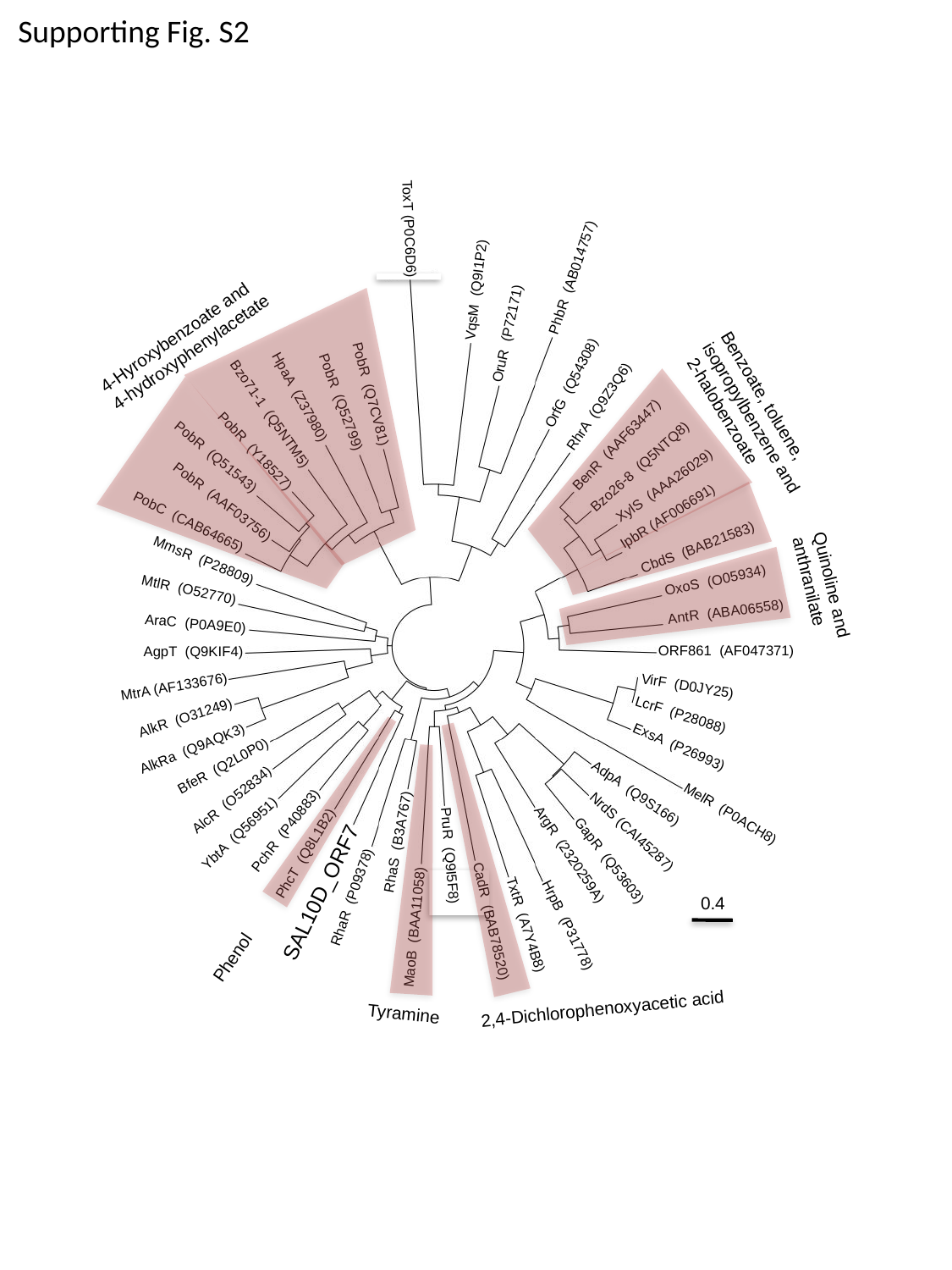

Supporting Fig. S2
PhbR (AB014757)
VqsM (Q9I1P2)
ToxT (P0C6D6)
OruR (P72171)
4-Hyroxybenzoate and
4-hydroxyphenylacetate
OrfG (Q54308)
RhrA (Q9Z3Q6)
Benzoate, toluene, isopropylbenzene and
 2-halobenzoate
BenR (AAF63447)
PobR (Q52799)
PobR (Q7CV81)
HpaA (Z37980)
Bzo71-1 (Q5NTM5)
Bzo26-8 (Q5NTQ8)
PobR (Q51543)
PobR (Y18527)
XylS (AAA26029)
IpbR (AF006691)
PobR (AAF03756)
PobC (CAB64665)
CbdS (BAB21583)
OxoS (O05934)
MmsR (P28809)
Quinoline and anthranilate
MtlR (O52770)
AntR (ABA06558)
AraC (P0A9E0)
ORF861 (AF047371)
AgpT (Q9KIF4)
MtrA (AF133676)
VirF (D0JY25)
AlkR (O31249)
LcrF (P28088)
AlkRa (Q9AQK3)
ExsA (P26993)
BfeR (Q2L0P0)
AlcR (O52834)
AdpA (Q9S166)
YbtA (Q56951)
PchR (P40883)
MelR (P0ACH8)
RhaS (B3A767)
PhcT (Q8L1B2)
NrdS (CAI45287)
SAL10D_ORF7
ArgR (2320259A)
RhaR (P09378)
GapR (Q53603)
PruR (Q9I5F8)
0.4
MaoB (BAA11058)
Phenol
HrpB (P31778)
CadR (BAB78520)
TxtR (A7Y4B8)
2,4-Dichlorophenoxyacetic acid
Tyramine
