# Supplementary material for: Metagenomic Screening for Aromatic Compound-Responsive Transcriptional Regulators
Source: PLoS One. 2013 Sep 30;8(9):e75795. doi: 10.1371/journal.pone.0075795 (PMC3786939; doi:10.1371/journal.pone.0075795)
Supplement: Figure S3 — Phylogenetic relationship between functionally characterized two component -type transcriptional regulators and our metagenomically retrieved homologues. A: Response regulator domain; B: histidine kinase domain. Shaded clones are known to be involved in the degradation of aromatic compounds. (PPTX) [file pone.0075795.s003.pptx]

## Slide 1
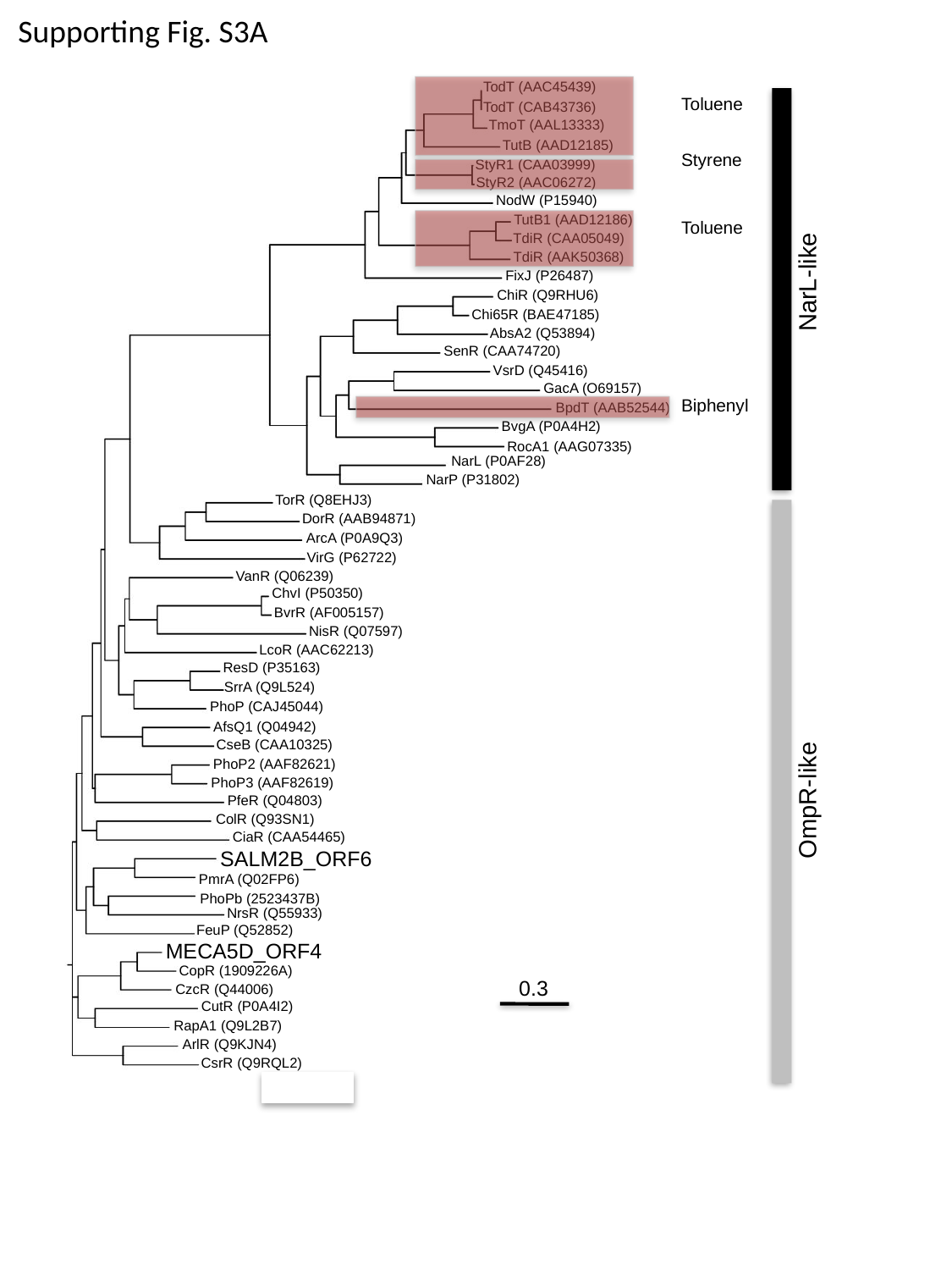

Supporting Fig. S3A
TodT (AAC45439)
Toluene
TodT (CAB43736)
TmoT (AAL13333)
TutB (AAD12185)
Styrene
StyR1 (CAA03999)
StyR2 (AAC06272)
NodW (P15940)
TutB1 (AAD12186)
Toluene
TdiR (CAA05049)
TdiR (AAK50368)
FixJ (P26487)
NarL-like
ChiR (Q9RHU6)
Chi65R (BAE47185)
AbsA2 (Q53894)
SenR (CAA74720)
VsrD (Q45416)
GacA (O69157)
Biphenyl
BpdT (AAB52544)
BvgA (P0A4H2)
RocA1 (AAG07335)
NarL (P0AF28)
NarP (P31802)
TorR (Q8EHJ3)
DorR (AAB94871)
ArcA (P0A9Q3)
VirG (P62722)
VanR (Q06239)
ChvI (P50350)
BvrR (AF005157)
NisR (Q07597)
LcoR (AAC62213)
ResD (P35163)
SrrA (Q9L524)
PhoP (CAJ45044)
AfsQ1 (Q04942)
CseB (CAA10325)
PhoP2 (AAF82621)
PhoP3 (AAF82619)
OmpR-like
PfeR (Q04803)
ColR (Q93SN1)
CiaR (CAA54465)
SALM2B_ORF6
PmrA (Q02FP6)
PhoPb (2523437B)
NrsR (Q55933)
FeuP (Q52852)
MECA5D_ORF4
CopR (1909226A)
0.3
CzcR (Q44006)
CutR (P0A4I2)
RapA1 (Q9L2B7)
ArlR (Q9KJN4)
CsrR (Q9RQL2)

## Slide 2
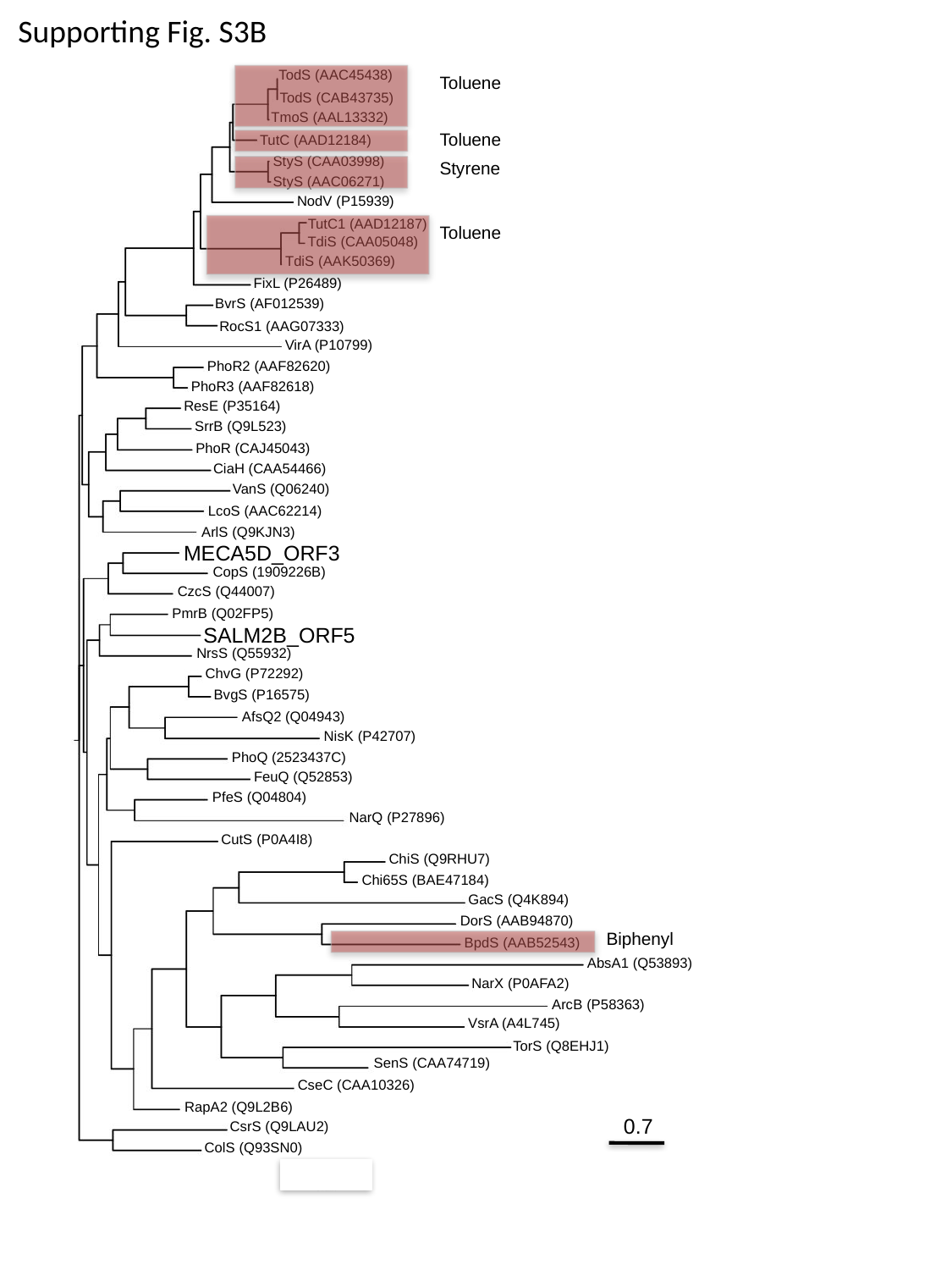

Supporting Fig. S3B
TodS (AAC45438)
Toluene
TodS (CAB43735)
TmoS (AAL13332)
Toluene
TutC (AAD12184)
StyS (CAA03998)
Styrene
StyS (AAC06271)
NodV (P15939)
TutC1 (AAD12187)
Toluene
TdiS (CAA05048)
TdiS (AAK50369)
FixL (P26489)
BvrS (AF012539)
RocS1 (AAG07333)
VirA (P10799)
PhoR2 (AAF82620)
PhoR3 (AAF82618)
ResE (P35164)
SrrB (Q9L523)
PhoR (CAJ45043)
CiaH (CAA54466)
VanS (Q06240)
LcoS (AAC62214)
ArlS (Q9KJN3)
MECA5D_ORF3
CopS (1909226B)
CzcS (Q44007)
PmrB (Q02FP5)
SALM2B_ORF5
NrsS (Q55932)
ChvG (P72292)
BvgS (P16575)
AfsQ2 (Q04943)
NisK (P42707)
PhoQ (2523437C)
FeuQ (Q52853)
PfeS (Q04804)
NarQ (P27896)
CutS (P0A4I8)
ChiS (Q9RHU7)
Chi65S (BAE47184)
GacS (Q4K894)
DorS (AAB94870)
Biphenyl
BpdS (AAB52543)
AbsA1 (Q53893)
NarX (P0AFA2)
ArcB (P58363)
VsrA (A4L745)
TorS (Q8EHJ1)
SenS (CAA74719)
CseC (CAA10326)
RapA2 (Q9L2B6)
0.7
CsrS (Q9LAU2)
ColS (Q93SN0)
