# Supplementary material for: Metagenomic Screening for Aromatic Compound-Responsive Transcriptional Regulators
Source: PLoS One. 2013 Sep 30;8(9):e75795. doi: 10.1371/journal.pone.0075795 (PMC3786939; doi:10.1371/journal.pone.0075795)
Supplement: Table S1 — Plasmids used in this study. (DOCX) [file pone.0075795.s004.docx]

**Supporting Table S1. Plasmids used in this study**

| Plasmids | Relevant characteristics | Sources |
| --- | --- | --- |
| pUC18 | Cloning vector | TaKaRa Bio |
| p18GFP | pUC18 containing promoter-less *gfp* | Uchiyama et al., 2005 |
| pSAL1A | p18GFP derivative carrying salicylate–inducible transcriptional regulator | This study |
| pSAL1H | p18GFP derivative carrying salicylate–inducible transcriptional regulator | This study |
| pSAL6F | p18GFP derivative carrying salicylate–inducible transcriptional regulator | This study |
| pSAL7A | p18GFP derivative carrying salicylate–inducible transcriptional regulator | This study |
| pSAL7A/EcoRV | pSAL7A derivative with 2.6 kb deletion at the *Eco*RV site | This study |
| pSAL7A/SacI | pSAL7A derivative with 3.0 kb deletion at the *Sac*I site | This study |
| pSAL10D | p18GFP derivative carrying salicylate–inducible transcriptional regulator | This study |
| pSAL10D/EcoRI | pSAL10D derivative with 6.9 kb deletion at the *Eco*RI site | This study |
| pSAL10D/SalI | pSAL10D derivative with 7.6 kb deletion at the *Sal*I site | This study |
| pSALM2B | p18GFP derivative carrying salicylate/3-methylcatechol–inducible transcriptional regulator | This study |
| pSALM2B/BsmI | pSALM2B derivative with 2.6 kb deletion at the *BsmI* site | This study |
| pSALM2B/XhoI | pSALM2B derivative with 3.8 kb deletion at the *Xho*I site | This study |
| pMECA2G | p18GFP derivative carrying 3-methylcatechol–inducible transcriptional regulator | This study |
| pMECA2G/SmaI | pMECA2G derivative with 2.2 kb deletion at the *Sma*I site | This study |
| pMECA2G/HincII | pMECA2G derivative with 5.0 kb deletion at the *Hin*cII site | This study |
| pMECA5D | p18GFP derivative carrying 3-methylcatechol–inducible transcriptional regulator | This study |
| pMECA5D/SacI | pMECA5D derivative with 1.2 kb deletion at the *Sac*I site | This study |
| pMECA5D/SphI | pMECA5D derivative with 2.4 kb deletion at the *Sph*I site | This study |
| pMECA7B | p18GFP derivative carrying 3-methylcatechol/4-methylcatechol–inducible transcriptional regulator | This study |
| pMECA7B/EcoRV | pMECA7B derivative with 6.8 kb deletion at the *Eco*RV site | This study |
| pMECA7B/XhoI | pMECA7B derivative with 7.2 kb deletion at the *Xho*I site | This study |
| pCHLO4C | p18GFP derivative carrying 4-methylcatechol–inducible transcriptional regulator | This study |
| pCHLO4C/SalI | pCHLO4C derivative with 3.1 kb deletion at the *Sal*I site | This study |
| pCHLO4C/SmaI | pCHLO4C derivative with 4.0 kb deletion at the *Sma*I site | This study |
| pCHLO6C | p18GFP derivative carrying 4-methylcatechol–inducible transcriptional regulator | This study |
| pCHLO6C/NotI | pCHLO6C derivative with 4.7 kb deletion at the *Not*I site | This study |
| pCHLO6C/Eco52I | pCHLO6C derivative with 5.6 kb deletion at the *Eco*52I site | This study |
| pCHLO8F | p18GFP derivative carrying 4-methylcatechol–inducible transcriptional regulator | This study |
| pCHLO8F/HincII | pCHLO8F derivative with 6.3 kb deletion at the *Hin*cII site | This study |
