# Supplementary material for: Metagenomic Screening for Aromatic Compound-Responsive Transcriptional Regulators
Source: PLoS One. 2013 Sep 30;8(9):e75795. doi: 10.1371/journal.pone.0075795 (PMC3786939; doi:10.1371/journal.pone.0075795)
Supplement: Table S2 — List of ORFs in metagenomically retrieved clones that fluoresced in response to aromatic compounds. Putative transcriptional regulators are shown in boldface and those responsible for compound-specific regulation are underlined. (DOCX) [file pone.0075795.s005.docx]

**Supporting Table S2.** List of ORFs in metagenomically retrieved clones that fluoresced in response to aromatic compounds. Putative transcriptional regulators are shown in boldface and those responsible for compound-specific regulation are underlined.

| Clone (bp) | ORF (aa) | Closest homologue (accession number) | Organism | Identity |
| --- | --- | --- | --- | --- |
| SAL1A  (10,899) | ORF1 (258, partial) | Nucleoside recognition domain-containing protein (YP_001792713) | *Leptothrix cholodnii* SP-6 | 77% (199/258) |
|  | ORF2 (342) | Aldo/keto reductase (YP_982255) | *Polaromonas naphthalenivorans* CJ2 | 76% (245/319) |
|  | ORF3 (483) | Aldehyde dehydrogenase (YP_001789841) | *Leptothrix cholodnii* SP-6 | 80% (388/482) |
|  | ORF4 (584) | Glucose-methanol-choline oxidoreductase (YP_001789840) | *Leptothrix cholodnii* SP-6 | 75% (424/564) |
|  | **ORF5 (403)** | **Fis family transcriptional regulator (YP_001789839)** | ***Leptothrix cholodnii* SP-6** | **49% (203/410)** |
|  | **ORF6 (305)** | **LysR-like regular protein (AAG13636)** | ***Ralstonia* sp. U2** | **78% (237/301)** |
|  | ORF7 (329) | Oxidoreductase component of 2,4-dinitrotoluene dioxygenase DntAa (AAL50024) | *Burkholderia cepacia* | 78% (258/329) |
|  | ORF8 (423) | Salicylate-5-hydroxylase large oxygenase component (AAD12607) | *Ralstonia* sp. U2 | 91% (389/423) |
|  | ORF9 (161) | Salicylate-5-hydroxylase small oxygenase component (AAD12608) | *Ralstonia* sp. U2 | 82% (133/161) |
|  | ORF10 (94, partial) | Ferredoxin (YP_982711) | *Polaromonas naphthalenivorans* CJ2 | 82% (78/94) |
| SAL1H  (11,513) | ORF1 (179, partial) | Putative substrate-binding component of ABC transporter (YP_002947964) | *Variovorax paradoxus* S110 | 77% (123/159) |
|  | ORF2 (432) | Putative substrate-binding component of ABC transporter (YP_002947963) | *Variovorax paradoxus* S110 | 55% (218/393) |
|  | ORF3 (265) | Hypothetical protein (YP_776197) | *Burkholderia ambifaria* AMMD | 70% (178/254) |
|  | ORF4 (483) | Aldehyde dehydrogenase (YP_001789841) | *Leptothrix cholodnii* SP-6 | 80% (387/482) |
|  | ORF5 (584) | Glucose-methanol-choline oxidoreductase (YP_001789840) | *Leptothrix cholodnii* SP-6 | 74% (422/564) |
|  | **ORF6 (403)** | **Fis family transcriptional regulator (YP_001789839)** | ***Leptothrix cholodnii* SP-6** | **49% (203/410)** |
|  | **ORF7 (305)** | **LysR-like regular protein (AAG13636)** | ***Ralstonia* sp. U2** | **78% (237/301)** |
|  | ORF8 (329) | Oxidoreductase component of 2,4-dinitrotoluene dioxygenase, DntAa (AAL50024) | *Burkholderia cepacia* | 79% (260/329) |
|  | ORF9 (407, partial) | Salicylate-5-hydroxylase large oxygenase component (AAD12607) | *Ralstonia* sp. U2 | 91% (375/410) |
| SAL6F  (8,089) | ORF1 (269, partial) | Meta-pathway phenol degradation-like protein (YP_373911) | *Burkholderia* sp. 383 | 53% (90/169) |
|  | ORF2 (584) | Glucose-methanol-choline oxidoreductase (YP_001789840) | *Leptothrix cholodnii* SP-6 | 74% (418/564) |
|  | ORF3 (358) | Hypothetical protein (YP_002890764) | *Thauera* sp. MZ1T | 55% (105/190) |
|  | **ORF4 (408)** | **Fis family transcriptional regulator (YP_001789839)** | ***Leptothrix cholodnii* SP-6** | **50% (205/408)** |
|  | **ORF5 (305)** | **LysR-like regular protein (AAG13636)** | ***Ralstonia* sp. U2** | **78% (235/301)** |
|  | ORF6 (329) | Oxidoreductase component of 2,4-dinitrotoluene dioxygenase, DntAa (AAL50024) | *Burkholderia cepacia* | 77% (254/329) |
|  | ORF7 (304, partial) | Salicylate-5-hydroxylase large oxygenase component (AAD12607) | *Ralstonia* sp. U2 | 91% (280/307) |
| SAL7A  (3,901) | ORF1 (358) | Hypothetical protein (YP_002890764) | *Thauera* sp. MZ1T | 55% (105/190) |
|  | **ORF2 (408)** | **Fis family transcriptional regulator (YP_001789839)** | ***Leptothrix cholodnii* SP-6** | **50% (205/408)** |
|  | **ORF3 (305)** | **LysR-like regular protein (AAG13636)** | ***Ralstonia* sp. U2** | **78% (235/301)** |
|  | ORF4 (64, partial) | Oxidoreductase component of 2,4-dinitrotoluene dioxygenase, DntAa (AAL50024) | *Burkholderia cepacia* | 73% (46/63) |
| SAL10D  (8,862) | ORF1 (450, partial) | Putative acetolactate synthase large subunit (YP_350199) | *Pseudomonas fluorescens* Pf0-1 | 92% (414/450) |
|  | ORF2 (486) | Betaine-aldehyde dehydrogenase (YP_350198) | *Pseudomonas fluorescens* Pf0-1 | 90% (436/482) |
|  | ORF3 (384) | Putative substrate-binding component of ABC transporter (YP_350197) | *Pseudomonas fluorescens* Pf0-1 | 95% (350/367) |
|  | ORF4 (302) | Putative ABC transport system, membrane protein (YP_350196) | *Pseudomonas fluorescens* Pf0-1 | 94% (286/302) |
|  | ORF5 (282) | Putative ABC transporter permease (YP_350195) | *Pseudomonas fluorescens* Pf0-1 | 96% (272/282) |
|  | ORF6 (351) | ABC transporter, nucleotide binding/ATPase protein (YP_350194) | *Pseudomonas fluorescens* Pf0-1 | 96% (339/351) |
|  | **ORF7 (310)** | **AraC family transcriptional regulator (YP_350193)** | ***Pseudomonas fluorescens* Pf0-1** | **93% (288/309)** |
|  | ORF8 (78, partial) | Antibiotic biosynthesis monooxygenase (YP_350192) | *Pseudomonas fluorescens* Pf0-1 | 91% (71/78) |
| SALM2B  (6,397) | ORF1 (153, partial) | Glyoxalase family protein (YP_261260) | *Pseudomonas fluorescens* Pf-5 | 65% (97/149) |
|  | ORF2 (399) | Putative membrane protein (YP_349651) | *Pseudomonas fluorescens* Pf0-1 | 77% (307/397) |
|  | ORF3 (180) | Hypothetical protein (YP_349652) | *Pseudomonas fluorescens* Pf0-1 | 72% (130/180) |
|  | ORF4 (105) | Hypothetical protein (YP_349653) | *Pseudomonas fluorescens* Pf0-1 | 91% (77/84) |
|  | **ORF5 (440)** | **Two-component system sensor histidine kinase, ArmS (AAF80269)** | ***Pseudomonas* sp. JR1** | **89% (395/440)** |
|  | **ORF6 (226)** | **Two-component system response regulator, ArmR (AAF80268)** | ***Pseudomonas* sp. JR1** | **95% (216/226)** |
|  | ORF7 (91, partial) | Putative disulfide isomerase, DsbD (AAF80267) | *Pseudomonas* sp. JR1 | 91% (83/91) |
| MECA2G  (7,410) | ORF1 (437, partial) | TRAP transporter solute receptor TAXI family (YP_283776) | *Dechloromonas aromatica* RCB | 84% (370/437) |
|  | ORF2 (208) | Putative partition-related protein (YP_283777) | *Dechloromonas aromatica* RCB | 91% (190/207) |
|  | **ORF3** **(907)** | **PAS/PAC sensor hybrid histidine kinase** | ***Allochromatium vinosum* DSM 180** | **43% (379/876)** |
|  | **ORF4** **(303)** | **LysR family transcriptional regulator (YP_283779)** | ***Dechloromonas aromatica* RCB** | **74% (227/303)** |
|  | ORF5 (235) | Pirin, N-terminal (YP_283780) | *Dechloromonas aromatica* RCB | 83% (194/233) |
|  | ORF6 (184) | Flavodoxin/nitric oxide synthase (YP_283781) | *Dechloromonas aromatica* RCB | 87% (161/184) |
| MECA5D  (6,513) | ORF1 (116, partial) | Tn5044 transposase (ZP_06703987) | *Xanthomonas fuscans* subsp. *aurantifolii* str. ICPB 11122 | 47% (55/115) |
|  | ORF2 (428) | Phage integrase (YP_549326) | *Polaromonas* sp. JS666 | 52% (231/440) |
|  | **ORF3** **(417)** | **Heavy metal sensor kinase (YP_285465)** | ***Dechloromonas aromatica* RCB** | **59% (260/434)** |
|  | **ORF4** **(230)** | **Two component heavy metal response transcriptional regulator (YP_285466)** | ***Dechloromonas aromatica* RCB** | **88% (199/225)** |
|  | ORF5 (479) | Putative copper resistance-related lipoprotein (YP_001020830) | *Methylibium petroleiphilum* PM1 | 59% (278/464) |
|  | ORF6 (288, partial) | Multi-Cu oxidase (YP_001019692) | *Methylibium petroleiphilum* PM1 | 85% (218/256) |
| MECA7B  (8,709) | ORF1 (505) | Hypothetical protein (YP_286139) | *Dechloromonas aromatica* RCB | 77% (390/505) |
|  | ORF2 (586) | Hypothetical protein (YP_286138) | *Dechloromonas aromatica* RCB | 68% (229/333) |
|  | ORF3 (290) | Hypothetical protein (YP_286137) | *Dechloromonas aromatica* RCB | 79% (218/274) |
|  | ORF4 (874) | Hypothetical protein  (YP_286136) | *Dechloromonas aromatica* RCB | 82% (691/842) |
|  | **ORF5** **(302)** | **LysR family transcriptional regulator**  **(YP_159878)** | ***Aromatoleum aromaticum* EbN1** | **62% (188/302)** |
|  | ORF6 (192, partial) | Predicted nucleoside-diphosphate-sugar epimerase  (YP_159879) | *Aromatoleum aromaticum* EbN1 | 61% (36/59) |
| CHLO4C  (4,955) | ORF1 (189) | Copper/zinc superoxide dismutase  (YP_286143) | *Dechloromonas aromatica* RCB | 63% (94/147) |
|  | **ORF2** **(314)** | **LysR family transcriptional regulator**  **(YP_522088)** | ***Rhodoferax ferrireducens* T118** | **68% (211/310)** |
|  | ORF3 (90, partial) | Putative pirin protein  (ZP_01915815) | *Limnobacter* sp. MED105 | 71% (47/66) |
| CHLO6C  (6,531) | ORF1 (348, partial) | Cellulose synthase operon protein C (ZP_07231490) | *Pseudomonas syringae* pv. *tomato* Max13 | 61% (193/313) |
|  | ORF2 (211) | Cellulose biosynthesis protein (YP_787124) | *Bordetella avium* 197N | 62% (131/211) |
|  | ORF3 (222) | Cell morphology protein (ZP_06460591) | *Pseudomonas syringae* pv. *aesculi* str. NCPPB3681 | 70% (158/224) |
|  | ORF4 (471) | Cell morphology-related protein (YP_002869988) | *Pseudomonas fluorescens* SBW25 | 87% (414/471) |
|  | ORF5 (378) | Cell morphology protein (ZP_06459195) | *Pseudomonas syringae* pv. *aesculi* str. NCPPB3681 | 59% (192/322) |
|  | **ORF6** **(308)** | **LysR family transcriptional regulator (YP_345874)** | ***Pseudomonas fluorescens* Pf0-1** | **93% (286/305)** |
|  | ORF7 (102, partial) | Putative ABC transport system, permease (YP_002869825) | *Pseudomonas fluorescens* SBW25 | 82% (84/102) |
| CHLO8F  (6,934) | ORF1 (80) | DegT/DnrJ/EryC1/StrS aminotransferase (YP_001953498) | *Geobacter lovleyi* SZ | 85% (68/80) |
|  | ORF2 (456) | Replicative DNA helicase (YP_001953499) | *Geobacter lovleyi* SZ | 91% (418/456) |
|  | ORF3 (360) | Flagellar hook-associated protein 3 (YP_001953500) | *Geobacter lovleyi* SZ | 38% (139/363) |
|  | ORF4 (482) | Flagellar hook-associated protein, FlgK (YP_001953501) | *Geobacter lovleyi* SZ | 49% (242/486) |
|  | ORF5 (165) | FlgN family protein (YP_001953502) | *Geobacter lovleyi* SZ | 71% (118/165) |
|  | ORF6 (126) | Flagellar protein FlgJ-like protein (YP_001953503) | *Geobacter lovleyi* SZ | 78% (99/126) |
|  | ORF7 (363) | Flagellar P-ring protein (YP_001953504) | *Geobacter lovleyi* SZ | 83% (304/363) |
|  | ORF8(224) | Flagellar L-ring protein (YP_001953505) | *Geobacter lovleyi* SZ | 82% (185/224) |
